# Atypical and agressive presentation of gastric cancer in a patient with common variable disease

**DOI:** 10.1186/1939-4551-8-S1-A170

**Published:** 2015-04-08

**Authors:** Pablo Torres, Myrthes Toledo Barros, Fabiana Mascarenhas, Karla Boufleur, Carolina Tavares De Alcântara, Luiz Augusto Marcondes, Cristina Kokron, Giane Garcia, Leonardo Mendonça, Jorge Kalil, Octavio Grecco

**Affiliations:** 1 Hcfmusp, Brazil; 2 Hospital Das Clínicas -Faculdade De Medicina –USP, Brazil; 3 University of São Paulo, Brazil

## Background

In patients with common variable immunodeficiency (CVID) gastrointestinal disorders and malignancies occur higher than expected in the general population. These patients present risk of 50 times higher than the population (Kalha and Kellin, 2004) for development of gastric cancer, what reinforces the importance of screening for premature diagnosis and treatment.

## Methods

Review of clinical data from eletronic records were performed. The objective of this paper is present a case of a patient with CVID with an atypical and very aggressive presentation of gastric cancer.

## Results

M.S.S., 32 female patient, followed with CVID since 21 years old, was hospitalized with 30 days of cought, fever unresponsive to oral treatment. Despite intravenous broad-spectrum antibiotics, there was no clinical improvement. Thorax CT was made that incidentaly showed multiple liver abscesses and after that the patient developed porgressive ascitis. Investigation of origin of these abscesses evidentiated two gastric ulcers and one rectal ulcer. The pathologic analysis of gastric and rectum byopsis and cytologic analysis of ascitis liquid showed adenocarcinoma of stomach with peritoneal carcinomatosis. In a familial discussion about the prognosis of this patient were defined exclusive paliative care. On the 24th day of hospitalization the patient a natural evolution to death.

## Conclusions

High incidence of gastric cancer in CVID reinforce necessity of screening. In our department we sugest to patients realize anual endoscopy with biopsy and this seems to be effective to early diagnosis and treatment. Despite our screening this patient had an unusual and aggressive presentation.

## Consent

Written informed consent was obtained from the patient for publication of this abstract and any accompanying images. A copy of the written consent is available for review by the Editor of this journal.

